# Literature Review—Transthoracic Echocardiography, Computed Tomography Angiography, and Their Value in Clinical Decision Making and Outcome Predictions in Patients with COVID-19 Associated Cardiovascular Complications

**DOI:** 10.3390/ijerph20126123

**Published:** 2023-06-14

**Authors:** Jędrzej Warpechowski, Adam Olichwier, Aleksandra Golonko, Marcin Warpechowski, Robert Milewski

**Affiliations:** 1 Clinical Research Center, Medical University of Białystok, 15-089 Białystok, Poland or aolichwier2@unl.edu (A.O.); golonkoaleksandra@gmail.com (A.G.); 2 Department of Nutrition and Health Sciences, University of Nebraska–Lincoln, Lincoln, NE 65588, USA; 3 Department of Biostatistics and Medical Informatics, Medical University of Białystok, 15-089 Białystok, Poland; marcin.warpechowski@umb.edu.pl (M.W.); robert.milewski@umb.edu.pl (R.M.)

**Keywords:** COVID-19, echocardiography, computed tomography angiography, pulmonary embolism

## Abstract

The sudden outbreak of the COVID-19 pandemic posed a great threat to the world’s healthcare systems. It resulted in the development of new methods and algorithms for the diagnosis and treatment of both COVID-19 and its complications. Diagnostic imaging played a crucial role in both cases. Among the most widely used examinations are transthoracic echocardiography (TTE) and computed tomography angiography (CTA). Cardiovascular complications in COVID-19 are frequently associated with a severe inflammatory response, which results in acute respiratory failure, further leading to severe complications of the cardiovascular system. Our review aims to discuss the value of TTE and CTA in clinical decision making and outcome prediction in patients with COVID-19-associated cardiovascular complications. Our review revealed the high clinical value of various TTE findings and their association with mortality and the prediction of patients’ clinical outcomes, especially when used with other laboratory parameters. The strongest association between increased mortality and findings in TTE was observed for tachycardia and decreased left ventricular ejection fraction (odds ratio (OR) 24.06) and tricuspid annular plane systolic excursion/pulmonary artery systolic pressure ratio (TAPSE/PASP ratio) < 0.31 mm/mmHg (OR 17.80). CTA is a valuable tool in diagnosing COVID-19-associated pulmonary embolism, but its association with mortality and its predictive role should always be combined with laboratory findings and patients’ medical history. D-dimers > 3000 ng/mL were found as the strongest predictors of pulmonary embolism (PE) (OR 7.494). Our review indicates the necessity for an active search for cardiovascular complications in patients with severe COVID-19, as they are linked with an increased probability of fatal outcomes.

## 1. Introduction

The novel severe acute respiratory syndrome coronavirus (SARS-CoV-2) was first discovered in December 2019 in Wuhan, China, when several cases of atypical pneumonia of unknown origin were reported [1]. During the next few months, this new disease, called COVID-19, spread rapidly all around the world, with incidence and mortality rates exceeding any expectations. The outbreak of the pandemic of COVID-19 was an unprecedented challenge for people and healthcare systems all around the world that also caused changes in our current lifestyle by introducing various types of restrictions or minimizing interpersonal contact [2]. In addition to many deaths caused by SARS-CoV-2, some convalescents suffer from various types of complications after being infected with the virus [3]. The physicians and scientists were confronted with the urgent necessity of developing methods and algorithms for the diagnosis and treatment of both COVID-19 itself and its long-term health consequences. The crucial factor in determining the severity of the disease as well as its complications is diagnostic imaging [4]. Among widely used examination methods in patients diagnosed with SARS-CoV-2 infection are transthoracic echocardiography (TTE) and computed tomography angiography (CTA). These methods are particularly valuable in establishing the lung surface affected by the disease or the presence of cardiovascular complications, such as pulmonary embolism or heart failure, which are among the most common outcomes of COVID-19 [5]. Diagnostic imaging, if combined with clinical and laboratory findings, may also facilitate early diagnosis. Chest computed tomography (CT) findings include bilateral, peripheral, and basal predominant consolidation, ground-glass opacity, often of an extensive geographical distribution, or both, along with many other less common abnormalities [5]. These findings can also be noticed before the onset of symptoms, and their extent increases significantly during the first and second weeks of the disease and decreases continuously afterward [6]. The evolution of the CT imaging corresponds with the progression of the SARS-CoV-2 infection, which makes it a useful tool to assess the current stage of a patient’s condition, as well as to detect COVID-19 pneumonia in some as yet asymptomatic patients [6]. However, those findings are not specific to COVID-19, so the diagnosis should not be established based on diagnostic imaging with no other clinical symptoms or laboratory tests. Moreover, studies show that chest CT may have a prognostic role as a predictor of a higher risk of a severe COVID-19 outcome or death, after adjustment for clinical risk factors and age. They therefore help to identify patients who may benefit from more aggressive treatment [7]. Finally, chest CT has a crucial role in the diagnosis of one of the most common COVID-19 complications, which is pulmonary embolism (PE), as well as atherosclerosis, myocardial injury, or acute myocarditis [8]. Echocardiography also gives a non-invasive assessment of cardiovascular conditions, which allows the physicians to immediately initiate appropriate treatment and consequently significantly improve the patient’s prognosis [9]. Therefore, our review aims to discuss the value of TTE and CTA in clinical decision making and outcome prediction in patients infected with the novel coronavirus with cardiovascular complications. 

The main subject of research was the role of conventional echocardiography and CTA in the management and treatment of patients diagnosed with COVID-19. Additionally, we also reviewed the most common echocardiography outcomes in SARS-CoV-2 infection. Medical subject heading (MeSH) terms included ‘COVID-19’, ‘transthoracic echocardiography’, and ‘computed tomography angiography’. Only original research studies concerning over 100 study subjects were included in the literature review. Non-English publications, systematic reviews, and case reports were excluded from the review.

## 2. Cardiovascular Complications of COVID-19

Most COVID-19 cardiovascular manifestations are associated with a systemic inflammatory response, which leads to hypercoagulability and hypercytokinaemia [10]. From the pathophysiological point of view, an increase in proinflammatory cytokines such as interleukins 6 and 2 (IL-6 and IL-2), but also tumor necrosis factor-alpha (TNF-α), causes damage to the endothelium, which further generates coagulation cascade, which is the key cause of disseminated intravascular coagulation (DIC), thromboembolic events, or bleeding [10]. However, acute respiratory failure caused by SARS-CoV-2 pneumonia also leads to impairment of the cardiovascular system, as respiratory and cardiovascular systems cooperate to maintain systemic homeostasis [10]. 

Myocarditis is an inflammation of the myocardium most often caused by infectious agents [11]. The exact mechanism is yet to be elucidated, but the systemic inflammatory response and replication and dissemination of SARS-CoV-2 are presumed to play an important role in myocardial damage [12]. This results in a reduction in the strength of myocardium contractility, which may finally result in acute heart failure [13]. Inflammation of the myocardium might also be complicated by pericarditis, which leads to pericardial effusion and can further result in cardiac tamponade [14].

Heart failure is one of the most important causes of mortality among COVID-19 patients and occurs as a direct myocardial injury caused by the activity of the coronavirus [15]. Myocardial damage is likely to be caused by viral interaction with spike 1 glycoprotein, which activates serine 2 transmembrane protease, resulting in myocardial dysfunction [15]. Respiratory failure leads to acute respiratory distress syndrome (ARDS), which further results in pulmonary hypertension and dysfunction of the right ventricle (RV), whereas septic shock, renal impairment, and volume increase are rather a cause of impairment of LV function [16]. Moreover, heart failure might also be a complication of myocarditis [15].

Thrombotic manifestations of SARS-CoV-2 occur often due to a severe inflammatory response, which leads to vascular damage [17]. A prothrombotic state leads to various thrombotic complications including deep vein thrombosis (DVT), pulmonary embolism (PE), or arterial thrombosis [18]. Less common COVID-19-associated cardiovascular manifestations include acute myocardial infarction (AMI) [11], arrhythmias [19], or takotsubo cardiomyopathy [19]. AMI and takotsubo cardiomyopathy are likely to be linked with myocardial damage, a generalized inflammatory response, and sympathetic activation caused by viral infection [16]. On the other hand, arrhythmias may occur due to the treatment of suspected viral infection [16]. Therefore, it is extremely important to develop methods for the early diagnosis of cardiovascular complications caused by COVID-19.

## 3. Diagnosis of COVID-19 Cardiovascular Complications

On physical examination, symptoms of cardiovascular manifestations might be difficult to distinguish from one other, as a majority of patients present with chest pain, dyspnea, cough, and fever [20], but in some cases, also with syncope, palpitations, chest discomfort, or post-exertional fatigue [21]. Nevertheless, those symptoms should be used to define the severity of myocarditis [22]. The presence of the symptoms mentioned should be further examined with the use of other diagnostic tools, including electrocardiography (ECG), the measurement of cardiac troponin (cTn), and echocardiography [22]. The presence of an elevation of the ST-segment (the interval between depolarization and repolarization of the ventricles) with no reciprocal ST-segment depression, an increased QRS complex (depolarization of ventricles) duration or a diffuse inversion of the T-wave (repolarization of the ventricular myocardium), the elevation of cTn, and abnormalities of ventricular motion in echocardiography indicate a possible inflammatory process of the myocardium [22]. Further steps should include cardiac magnetic resonance (CMR) and a biopsy of the myocardium [22].

The diagnosis of SARS-CoV-2 associated heart failure is similar, as it also requires a physical examination, the measurement of laboratory parameters such as atrial natriuretic peptide (ANP) and B-type natriuretic peptide (BNP), and an examination of heart condition via ECG and echocardiography [16]. In addition, laboratory examinations should include natriuretic peptides, and cardiac function should be further monitored using echocardiography [16]. Moreover, the diagnosis of COVID-19-associated PE is also difficult. COVID-19 pneumonia might diminish PE symptoms, as typically, reported dyspnea and hypoxemia are likely to occur also during viral pneumonia [23]. Clinical probability scores such as the Wells score may be a support tool, but it is important to remember that underestimation of the COVID-19-associated PE probability is possible [24]. D-dimer is also used to assess clinical probability, but it is not clear if its value can definitely “rule out” or “rule in” PE probability [25]. Therefore, CTA should be regarded as a first-choice method due to its availability and accuracy [23]. 

## 4. TTE in COVID-19 Cardiovascular Complications

Many studies are focused on using TTE as a method for COVID-19 complication detection. Therefore, we try to summarize and systematize the current state of knowledge in this field in Table 1 and the detailed descriptions below. 

A prospective international study that consisted of 1216 patients [9] showed that in 405 patients, abnormal echocardiographic findings led to a change in patient management. Abnormalities in echocardiograms were found in 667 patients. Of these, 305 were diagnosed with left ventricle (LV) abnormalities and 185 with RV abnormalities, and 174 had abnormalities in both ventricles [9]. In addition, abnormalities were also more common among patients with an ST-segment elevation in their ECG or increased biomarkers of cardiac function [9]. The study, which included 1000 patients, showed that a severe or moderate course of COVID-19 is more associated with significantly impaired echocardiographic parameters than a mild course of the infection [26]. During 3 months’ follow-up, echocardiography showed a significant change in LV internal dimension-diastole (LVIDd) (+1 ± 0.3 vs. +0.6 ± 0.3 mm) with a 95% confidence interval (CI) (0.32–1.67 and 0.09–1.29 respectively), left atrial (LA) volume (+7.7 ± 0.1 (CI 7.35–8.04) vs. +1.3 ± 0.1 (CI 0.99–1.61) mL/m^2^), LV ejection fraction (LVEF) (−3.8 ± 0.3 (CI 4.51–3.08) vs. −1.1 ± 0.3 (CI 1.89–0.30) %), RV internal dimension-diastole (RVIDd) (+2.2 ± 0.1 (CI 1.84–2.55) vs. +0.4 ± 0.1 mm (CI 0.12–0.87)), right atrium internal dimension (RAID basal) (+1.6 ± 0.1 (CI 1.22–1.97) vs. +0.4 ± 0.1 mm (95% CI 0.12–0.87)), tricuspid annular plane systolic excursion (TAPSE) (−2.7 ± 0.2 (CI 3.22–2.17) vs. −0.7 ± 0.2 mm (CI 1.26–0.13)), and tricuspid maximum velocity (Vmax) (+1.0 ± 0.1 (CI 0.79–1.20) vs. +0.3 ± 0.1 cm/s (CI 0.09–0.51)) in patients with moderate to severe and mild COVID-19, respectively [26]. 

An international multicenter study of 870 subjects found that approximately 30% of subjects were diagnosed with RV dysfunction and approximately 20% with LV dysfunction [27]. Mild LV dysfunction (LVEF, 40–50%) was noted in 11%, whereas moderate (LVEF, 30–40%) and severe (LVEF < 30%) were noted in 5% and 3%, respectively [27]. In addition, significant differences were also noted, as patients from Asia had better echocardiographic parameters, including LVEF, LV longitudinal strain (LVLS), RV free-wall strain (RVFWS), and RV basal diameter (RVBD), in comparison to subjects from the United States, Europe, and Latin America [27]; these parameters were also linked to mortality. A retrospective analysis of 724 hospitalized patients with a minimum of one echocardiographic examination [28] showed LV diastolic dysfunction in 20% and RV systolic dysfunction defined as RVFWS < 20% in 16% of hospitalized subjects but did not report any significant differences between intensive care unit (ICU) and non-ICU patients [28]. The prediction of fatal outcomes using a receiver operator characteristics (ROC) curve resulted in their increase after the addition of echocardiographic measurements (a rise from AUC = 0.77 (CI 0.70–0.84) to AUC = 0.91 (CI 0.85–0.96)) [28]. 

A cross-sectional study with 680 hospitalized COVID-19 patients [29] found that LVEF < 30%, pleural effusion, pulmonary artery systolic blood pressure (PASP) from 35 to 50 mmHg, RV dysfunction, and collapsed inferior vena cava (IVC) are independent risk factors for in-hospital mortality with a relative risk (RR) of 1.19 (CI 1.07–1.32), 1.08 (CI 1.00–1.16), 1.11 (CI 1.03–1.18), 1.54 (CI 1.40–1.08), and 1.05 (CI 1.01–1.08), respectively [29]. Retrospective international research conducted on 677 ICU patients [30] found RV systolic dysfunction among 152 subjects; LV systolic dysfunction was found among 149 patients [30], and a link between LV systolic dysfunction and increased mortality was also reported, with an odds ratio (OR) of 1.52 (CI 1.04–2.23)).

Three studies, which included 531 [31], 530 [32], and 510 [33] hospitalized patients with SARS-CoV-2 infection, reported enlargement of LA (20%) and RV (35%), decreased TAPSE (27%), and elevated systemic vascular resistance (SVR) index (61%). Patients with a low SVR index were more likely to develop severe COVID-19 infection, and an abnormal LV stroke work index (LVSWI) was associated with increased mortality (43% vs. 19%) [31]. The second study reported pericardial effusion, and its association with mortality, in 14% of patients (OR 1.83, (CI 0.95–3.4)), TAPSE, and LVEF were also linked to increased fatal outcomes (OR 2.3, (CI 1.39–3.65)) [32]. The third study reported an increased prevalence of RV dilatation (35%) and dysfunction (15%) and their independent roles in an augmented probability of death [33]. 

Studies that consisted of 301–500 patients reported that enlargement of the RV in TTE was more prevalent in patients with shock (OR 1.81, CI 1.03–3.18), thromboembolic events (OR 2.31, CI 1.37–3.88), the need for renal replacement therapy (RRT) (OR 2.35, CI 1.31–4.21), or a fatal outcome at 60 days (OR 1.93, CI 1.13–3.30), and abnormal RV function was more often linked to shock (OR 1.75, CI 1.21–2.92) [34]. RV dilatation was significantly associated with patients’ primary composite outcome (ICU or death) (*p* = 0.03) [35]. First-phase EF < 25% was found to be a strong predictor of death due to the increased mortality of subjects with first-phase EF < 25% (35.7%) in comparison to subjects with first-phase EF > 25% (7.8%) [36]. RV dysfunction, LV wall abnormalities, LV global dysfunction, diastolic dysfunction grade II or III, and pericardial effusion were more common in patients with elevated biomarkers of myocardial injury than in subjects without elevation (26.9% vs. 10.7%, 23.7% vs. 4.4%, 18.4% vs. 7.8%, 22.3% vs. 2.4%, and 10.6% vs. 1.8%, respectively) [37]. The observational study divided patients into three classes: first, normal RV function (52%), second, dilated RV with mostly preserved systolic function (31%), and third, RV dilatation with systolic impairment (17%), which showed a significant difference in 90-days mortality, outcome, and response to mechanical ventilation: 22%, 42%, and 73%, respectively, *p*-value (*p* < 0.001) [38]. RV dysfunction in TTE and troponin levels were also found to be good predictors of pulmonary embolism (PE) (AUC = 0.77) [39]. A retrospective study of 368 subjects found a significant association between LA dilation and LV thrombus in patients with ischemic stroke in comparison to subjects without (48.3% vs. 27.9%, *p* = 0.04; 4.2% vs. 0.7%, *p* = 0.03 respectively) [40]. The OR of the COVID-19 ischemic stroke risk, which also consisted of the findings mentioned, was 4.1 (CI 1.40–16.10), and AUC = 0.70 [40].

Studies of 201–300 patients found a significant decrease in mean LVEF in deceased (25 ± 12.38) and ICU patients (25.31 ± 11.89) compared to survivors (36.98 ± 12.7) and non-ICU patients (36.96 ± 12.72). Moreover, a significant association of tachycardia and LVEF with mortality (OR 24.06, CI 4.63–125.11) was also reported [41]. Pulmonary artery systolic pressure (PASP) in TTE was significantly linked to mortality in ICU patients (OR 1.09, CI 1.06–1.13), and TAPSE (OR 0.8, CI 0.72–0.88), TAPSE/PASP (0.05 × 10^−1^ [0.08 × 10^−2^, 0.03], CI 0.001–0.09), and PASP (OR 1.08, CI 1.04–1.12) were also more associated with developing PE [42]. The association of reduced LVEF ≤ 50%, TAPSE ≤ 17 mm, and the presence of ARDS was also found to increase fatal outcomes [43]. Mildly reduced RV systolic function (OR 3.51), moderately to severely reduced RV function (OR 7.3, CI 2.20–24.25), pulmonary hypertension (OR 5.39, CI 1.96–14.86), and moderate to severe tricuspid regurgitation (OR 3.92, CI 1.71–9.03) were found to be linked with the mortality of COVID-19 patients, whereas moderately to severely reduced RV systolic function (OR 3.49, CI 1.08–11.29) and pulmonary hypertension (OR 3.96, CI 1.33–11.75) had higher odds for ventilator use [44]. Higher odds for vasopressor use were also found for mildly reduced RV systolic function (OR 2.26, CI 1.06–4.85) [44]. A higher risk of RR was reported for moderately to severely reduced RV systolic function (OR 3.69, CI 1.19–11.41) and pulmonary hypertension (OR 4.88, CI 1.69–14.09), moderately reduced LV systolic function (OR 4.17, CI 1.12–15.53), severely reduced LV systolic function (3.44), moderate to severe tricuspid regurgitation (OR 6.63, CI 2.58–17.02), mildly reduced RV systolic function (OR 7.71, CI 2.56–23.22), and enlarged RV (OR 3.11, CI 1.58–6.12) [44]. Patients with biventricular dysfunction had higher mortality in comparison to RV or LV dysfunction [45]. A significant link with in-hospital mortality was found for PASP > 35 mmHg (OR 5.82, CI 2.84–11.90), RV FS (OR 3.4, CI 1.25–9.18), TAPSE < 17 mm (OR 3.06, CI 1.30–9.18), RV S wave < 9.5 (OR 2.4, CI 0.99–5.83), and TAPSE/PASP < 0.31 mm/mmHg (OR 17.8, CI 3.70–86.31) [46]. 

Studies of 151–200 patients found LV hypertrophy or enlargement (77.5%), RV enlargement (59%), tricuspid regurgitation (54.5%), LVEF < 50% (50%), pulmonary hypertension (45.5%), and pericardial effusion (12.5%) but did not find any association between TTE findings and mortality [47]. On the other hand, a study with the same number of subjects found an association between abnormal TAPSE, hazard ratio (HR) (HR 4.3, CI 1.68–11.60), and stroke work index (SWI) (HR 2.12, CI 1.00–5.23) [48]. Pulmonary hypertension (PH) and RV dysfunction in TTE in COVID-19 subjects increased the probability of ICU admission or fatal outcome (41.7% vs. 8.5%). RVSP ≥35 mmHg was also helpful to stratify the short-term risk of fatal [49] outcomes with other parameters including D-dimer (small protein fragment made when a blood clot dissolves in the body), troponin, and prior cardiovascular disease (AUC = 0.81, CI 0.74–0.88) [50]. Patients with RV dilation also had a higher risk of death (49% vs. 33%) as well as patients with RV systolic impairment (53% vs. 28%) [51]. LVEF (OR = 0.95, CI 0.91–0.99; optimal cutoff: <64%) and TAPSE (OR = 0.76, CI 0.63–0.91; optimal cutoff: <18.5 mm) were found as predictors of mortality in hospitalized patients with COVID-19 [52]. Similar findings for LVEF (HR 0.955, CI 0.926–0.984) were also presented in other research, as well as a connection between tricuspid regurgitation (HR 2.851, CI 1.480–5.490) and impaired RV function (HR 2.463, CI 1.239–4.895) as independent poor outcome predictors [53]. Moreover, a similar HR value for LVEF and mortality was presented by another group (HR 0.94, CI 0.89–0.99), which additionally showed increased mortality association between cor pulmonale (in the structure and function of the RV) (HR 4.05, CI 1.09–15.02) and RV dilatation (HR 3.33, CI 1.29–8.61) [54]. 

In studies with 100–150 subjects, lower LVEF (50 ± 13 vs. 56 ± 11%), higher PASP (42 ± 12 vs. 34 ± 12 mmHg), decreased TAPSE (19 ± 4 vs. 22 ± 4 mm), and a lower TAPSE/PASP ratio (0.48 ± 0.18 vs. 0.72 ± 0.32 mm/mmHg) were discovered in SARS-CoV-2 non-survivors vs. survivors, but also a 27% lower risk of death for every 1 mm/mmHg increase in TAPSE/PASP. The TAPSE/PASP ratio cutoff for predicting mortality was found to be <0.57 mm/mmHg [55]. In an observational study, RV dysfunction (OR 7.03, 2.08–23.80) and elevated PASP (OR 3.88, CI 1.45–10.40) were associated with a higher risk of death at 30 days [56]. A higher proportion of ventricular abnormalities (38.5% vs. 18.4%) and lower TAPSE (1.8 ± 0.2 cm vs. 2 ± 0.3 cm) were observed in patients on ventilation support vs. patients without ventilation. A higher proportion of ventricular abnormalities (38.5% vs. 18.4%) and lower TAPSE (1.8 ± 0.2 cm vs. 2 ± 0.3 cm) were observed in patients on ventilation support vs. patients without ventilation support; an abnormal echocardiogram was associated with advanced ventilatory support (OR 4.83, CI 1.50–15.30), acute decompensated heart failure (OR 22.8, CI 2.6–200.4), myocardial injury (OR 5.19, CI 1.60–19.90), acute kidney injury (OR 5.46, CI 1.90–15.50), death (OR 6.82, CI 1.20–39.70), and composite endpoint (including death, mechanical ventilation, shock, and acute decompensated heart failure) (OR 7.29, CI 2.44–20.00) [57]. Intriguingly, there was also no association between TTE findings and the course of infection [58]. Another study did not report any correlation between TTE parameters and mortality, but tricuspid velocity was linked with PE, acute renal failure, or fatal outcome (*p* = 0.003, AUC = 0.739, CI 0.61–0.87) [59].

Some reports indicate that the use of invasive mechanical ventilation (IMV) might influence the results of TTE [60]. In studies that were utilized in our review, there was no information about TTE alterations among patients with advanced ventilatory support, but we found one study describing the possible impact of invasive mechanical ventilation on a lower prevalence of RV abnormalities [61]. 

**Figure 1:**
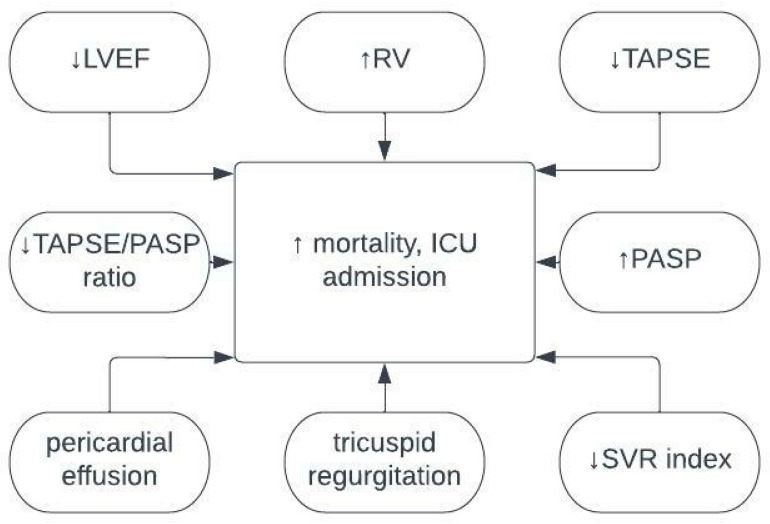
Association between echocardiography findings and endpoint outcomes. LVEF—left ventricle ejection fraction; PASP—pulmonary artery systolic pressure; RV—right ventricle; SVR—systemic vascular resistance; TAPSE—tricuspid annular plane systolic excursion.

To sum up, studies conducted on bigger cohorts indicate the precious clinical value of TTE in the clinical management of patient conditions, a higher prevalence of ventricular abnormalities in COVID-19-associated cardiovascular complications, and continental differences in the severity of cardiovascular complications, but also a less pronounced association between TTE findings and fatal outcomes. On the other hand, studies with smaller cohorts show a strong link between various TTE findings and mortality and the risk for ICU admission and/or ventilatory support, but they also report no association at all. Consistently across all studies, TTE was shown as a strong method that leads to the prediction of patients’ conditions, which can be used for the prediction of patients’ clinical outcomes, their risk of death, or changes in management. The summary of the studies discussed is presented in Table 1. Associations between TTE findings and endpoint outcomes are also presented in Figure 1.

## 5. CTA in COVID-19 Cardiovascular Complications

Another method most often used to observe COVID-19 cardiovascular complications is CTA. We summarize observed CTA changes in the working cardiovascular system in Table 2 and describe the available literature reports in more detail below.

A retrospective observational study of 1240 patients found 8.3% of patients were diagnosed with PE, but 77.7% were diagnosed using CTA within the first 48 h after admission [62]. However, another large-scale case-control study of 316 subjects including 158 individuals with confirmed SARS-CoV-2 infection suggested the overutilization of CTA in COVID-19-associated PE, as it was diagnosed in 8.9% of COVID-19 subjects in comparison to 39.9% of non-COVID-19 patients [63]. Another study with 413 subjects found PE among 25% of all subjects—29% of ICU vs. 24% of non-ICU patients—but did not report any significant difference in mortality between patients with and without PE [64]. A retrospective, multicenter, observational study with 399 patients diagnosed 22% with PE, with 32% of cases occurring among SARS-CoV-2 positive individuals, but no significant difference between radiologic, clinical, or laboratory parameters and their impact on outcomes was noted [65]. Another multicenter study detected PE in 33% of cases but did not find any significant difference in mortality [66]. Retrospective comparative research conducted on 300 subjects confirmed PE in 15% of patients, with bilateral involvement in 57%, highly sensitive cardiac troponin T (cTnT-hs) and N-terminal (NT)-pro hormone BNP (a non-active prohormone that is released from the same molecule that produces BNP) and significantly elevated in PE patients; D-dimer was elevated in all patients, with no significant difference between PE and non-PE patients. Age (OR 1.024, CI 1.002–1.047), D-dimers (1.018, CI 1.004–1.031), and cTnT-hs (OR 1.007, CI 1.002–1.012) were identified as PE predictors [67].

A study of 274 individuals confirmed PE in 25.54% of patients; D-dimer > 3000 ng/mL (OR 7.494, CI 3.038–18.485) and tachypnoea (OR 4.967, CI 2.053–12.018) were predictive factors of PE [68]. Studies with a similar number of patients [69,70] confirmed PE among 21.93% and 18.7% of patients, respectively. An increased risk of PE was observed in patients treated by IMV [69] (OR 8.07, CI 2.70–23.82), and higher mortality was seen in PE patients vs. non-PE patients (20.3% vs. 16.1%) [69]. Non-Caucasian race (OR 5.44) and previous venous thromboembolism (VTE) (OR 5.30, CI 1.09–26.17) were reported as independent PE risk factors [70]. A retrospective, multicenter, observational study of 214 patients hospitalized due to COVID-19 diagnosed PE in 14% [71]. Moreover, an association between the presence of PE and the time between symptom onset and hospitalization (OR 1.07, CI 1.03–1.11), acute cardiac injury (OR 2.25, CI 1.05–2.25), D-dimer (OR 1.02, CI 1.01–1.05), TAPSE (OR 0.84, CI 0.66–0.96 = 8), and PASP (OR 1.12, CI 1.03–1.23) were associated with higher mortality; in patients with PE fatal outcome was 50% vs. 27% in patients with no PE, and cardiogenic shock was diagnosed in 37% of patients with PE vs. 14% of patients with no PE [71].

A study with fewer than 200 patients reported an association between computed tomography severity score (CTSS) and the increased probability of ICU admission (OR 1.21, CI 1.10–1.34), and death (OR 1.15, CI 1.03–1.30) [72]. A multicenter study of 169 patients confirmed PE in 15.4%; median D-dimer was significantly higher in patients with PE vs. those without PE (9.84 mg/L vs. 1.64 mg/L) [73]. Another multicenter research study with a similar number of patients detected PE in 44.7%; a significant correlation between pulmonary artery obstruction (PAO) index and D-dimer level (*p* = 0.002) was reported, but without any significant differences between CTA or laboratory findings and patients’ outcomes [74]. CTA research on 100 subjects found 235 with acute PE. PE patients were more frequently hospitalized in ICU (74% vs. 29%) and required mechanical ventilation (65% vs. 25%) [75]. 

**Figure 2:**
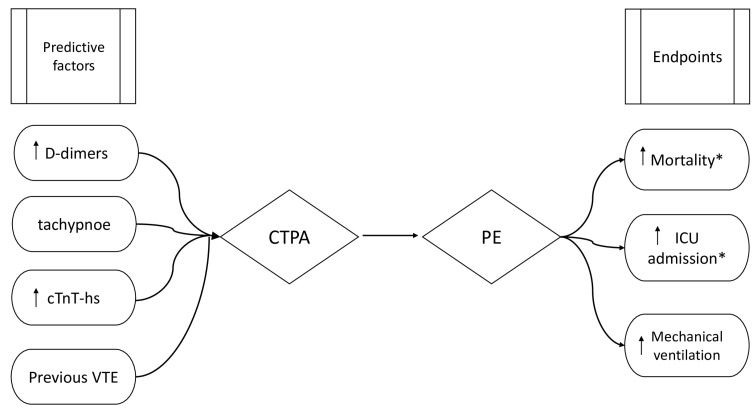
Predictive factors and endpoints of pulmonary embolism. The presence of predictive factors should be the indicator for the use of CTPA to fully confirm the pulmonary embolism. cTnT-hs—highly sensitive cardiac troponin T; VTE—venous thromboembolism; CTPA—computed tomography pulmonary angiogram; ICU—intensive care unit; PE—pulmonary embolism; *—increased probability when alterations on echocardiographic examinations are present.

In sum, CTA might be a valuable tool to confirm the diagnosis of COVID-19-associated PE, but some studies also report no association and the overutilization of CTA. Clinical outcomes, the mortality risk, and changes in the management of COVID-19-associated PE should always be analyzed with other clinical parameters, especially D-dimers, troponins, natriuretic peptides, or the patient’s medical history and general condition. The predictive factors and endpoints of PE are presented in Figure 2.

## 6. Conclusions and Prospects

The results presented in this review indicate that various parameters measured with TTE have prognostic value, which may influence the clinical decisions of physicians regarding patients’ management. The most frequently changed parameters detected with TTE belong to LV dysfunction measured as decreased values for LVEF, abnormal RV function, TAPSE, or TAPSE/PASP ratio. These changes are associated with the prediction of clinical outcomes and mortality. TAPSE/PASP < 0.31 mm/mmHg, as well as combined tachycardia and decreased LVEF, were reported as the most significant risk factors associated with increased mortality (OR 17.80, CI 3.70–86.31, and OR 24.06, CI 4.63–125.11, respectively). Nevertheless, TTE parameters alone or combined with patients’ medical history and laboratory parameters, including troponin, D-dimers, or natriuretic peptides, have high value as predictors of patients’ clinical outcomes in some cases. CTA is a valuable tool for making a firm diagnosis of PE, but clinical management and predictive value should be always combined with patients’ clinical history and/or laboratory parameters. D-dimers with a value over 3000 ng/mL were found to be the best predictive factor for PE (OR 7.494, CI 3.038–18.484), and IMV was reported as the most significant risk factor for PE (OR 8.07, CI 2.70–23.82). However, results obtained by CTA, such as fatal outcomes and an urgent need for ICU admission, can relate to D-dimers, cTnT-hs and NT-pro BNP, and CTA imaging findings. 

It is worth considering whether there is a necessity for an active search for thrombotic or cardiac problems in every patient previously hospitalized due to SARS-CoV-2 or only in patients with specific risk factors, and if so, what those risk factors should be. Another matter for discussion is which examination would be the best for screening for COVID-19 complications—many factors, such as sensitivity and specificity, costs, or the duration of such an examination, should be taken into consideration. For that reason, further research is required to establish whether there is an actual necessity for active screening for a post-COVID-19 complication such as pulmonary embolism, and if the answer to this question is positive, then one would need to decide which group of patients should be considered for such screening and which methods should be used. 

## 7. Limitations

The study is subject to several limitations, including the many retrospective studies used, which may be prone to selection and observer bias. Furthermore, the study population had varying stages of disease progression, coexisting conditions, and other risk factors, as well as specific conditions during the conduct of the research, especially in relation to TTE, which could have influenced the final outcome.

## Figures and Tables

**Table 1:** Summary of the studies on the value of echocardiography in COVID-19.

Author	Year	Population	Number of Patients	Key Observations
Dweck et al. [9]	2020	Patients with presumed or confirmed COVID-19 with indications for cardiac imaging	1216	Cardiac abnormalities found in TTE resulted in changing clinical management in 33% of patients
Chaturvedi et al. [26]	2022	Hospitalized patients with COVID-19	1000	Significantly greater change in echocardiographic parameters in patients with moderate to severe COVID-19 compared to patients with mild COVID-19
Karagodin et al. [27]	2021	Hospitalized patients with COVID-19	870	In a significant number of patients with RV dysfunction, impaired LVEF is more common among ICU patients
Pournazari et al. [28]	2021	Hospitalized patients with COVID-19	724	LV and RV dysfunction are more prevalent in hospital patients, with no significant differences between ICU and non-ICU patients, TTE findings as a predictor of mortality
Pishgahi et al. [29]	2021	Hospitalized patients with COVID-19	680	Decreased LVEF, pleural effusion, PASP, RV dysfunction, and collapsed IVC as risk factors for in-hospital mortality
Huang et al. [30]	2022	ICU patients with COVID-19	677	Association between LV systolic dysfunction and mortality
Taieb et al. [31]	2021	Hospitalized patients with COVID-19	531	Lower O_2_ saturation and higher prevalence of severe disease in patients with low SVR index compared to the patients with normal or increased SVR index in the high-risk MEWS group; mortality was higher in patients with abnormal LVSWI compared to patients with normal LVSWI
Ghantous et al. [32]	2022	Hospitalized patients with COVID-19	530	Pericardial effusion was associated with mortality, TAPSE, LVEF, and pericardial effusion associated with mortality
Kim et al. [33]	2020	Hospitalized patients with COVID-19	510	RV dilatation and dysfunction independently augmented the risk of mortality
Gomez et al. [34]	2022	Hospitalized patients with COVID-19	427	Thromboembolic events, need for RRT, and death at 60 days shock were linked to the increased probability of a fatal outcome
Soulat-Dufour et al. [35]	2022	Hospitalized patients with COVID-19	445	Primary composite outcome (ICU or death) associated with RV dilatation
Gu et al. [36]	2021	Hospitalized patients with COVID-19	380	First-phase EF < 25% was a strong predictor of death
Giustino et al. [37]	2020	Patients with confirmed COVID-19 who have undergone echocardiography during hospitalization	305	In patients with a biomarker of myocardial injury, RV dysfunction, LV wall abnormalities, LV global dysfunction, and diastolic dysfunction grade II or III, and pericardial effusion was more prevalent than in patients without increased biomarkers
Chotalia et al. [38]	2022	Hospitalized patients with COVID-19	305	90 days mortality significantly increased in dilated RV with preserved and impaired systolic function
Satoskar et al. [39]	2022	Hospitalized patients with COVID-19	302	A significant association between D-dimer > 5 mg/L and PE (OR 4.4), a predictive association of model with D-dimer > 5 mg/L, RV dysfunction, and troponin with PE
Oates et al. [40]	2022	Hospitalized patients with COVID-19	368	LA dilation and LV thrombus more prevalent in patients with ischemic stroke
Savarrakhsh et al. [41]	2022	Hospitalized patients with COVID-19	228	Mean LVEF was significantly lower in deceased and ICU patients Compared to survivors and non-ICU patients, with a significant association of tachycardia and LVEF with mortality
Polito et al. [42]	2021	Hospitalized patients with COVID-19	227	Association between TAPSE and TAPSE/PASP and mortality in ICU and non-ICU patients, an association between PASP and mortality in ICU patients, an association of TAPSE TAPSE/PASP (and PASP with developing PE)
Silverio et al. [43]	2021	Hospitalized patients with COVID-19	226	Association of reduced LVEF, TAPSE, and presence of ARDS with mortality
Wats et al. [44]	2021	Hospitalized patients with COVID-19	214	A significant association between mildly reduced RV systolic function, moderately to severely reduced RV function, pulmonary hypertension, and moderate to severe tricuspid regurgitation with mortality, association of moderately to severely reduced RV systolic function and pulmonary hypertension with higher odds for ventilator use, association of mildly reduced RV systolic function, moderately to severely reduced RV systolic function and pulmonary hypertension with higher odds for vasopressor use, association of moderately reduced LV systolic function, severely reduced LV systolic function, moderate to severe tricuspid regurgitation, mildly reduced RV systolic function, and enlarged RV with a higher risk of renal replacement therapy
Lassen et al. [45]	2020	Hospitalized patients with COVID-19, the control group matched on age, sex, and hypertension	214	Systolic function reduced in COVID-19 patients; reduced RV function higher mortality in patients with LV and RV dysfunction
Manzur-Sandoval et al. [46]	2021	Hospitalized patients with COVID-19	204	Association of PASP > 35 mmHg, RV FS; TAPSE < 17 mm, RV S wave < 9.5 and TAPSE/PASP < 0.31 mm/mmHg with in-hospital death
Meel et al. [47]	2021	Hospitalized patients with COVID-19	200	No statistically significant association between echocardiographic findings and mortality
Szekely et al. [48]	2021	Hospitalized patients with COVID-19	200	Abnormal TAPSE, LVEF, and SVI TAPSE and SVI were independent predictors of mortality
Pagnesi et al. [49]	2020	Patients with COVID-19 admitted to non-ICU departments	200	The rate of ICU admission or death was higher in patients with PH
Krishna et al. [50]	2021	Hospitalized patients with COVID-19	179	RVSP may be used in short-term risk stratification
Chotalia et al. [51]	2021	ICU patients with COVID-19	172	Increased mortality in patients with RV dilatation vs. patients without RV dilatation and patients with vs. without RV systolic impairment
Pimentel et al. [52]	2021	Hospitalized patients with COVID-19	163	Predictors of mortality: LVEF, TAPSE
Petersen-Uribe et al. [53]	2021	Hospitalized patients with COVID-19	157	Independent poor outcome predictors: tricuspid regurgitation, impaired RV function, decreased LVEF
Diaz et al. [54]	2022	ICU patients with COVID-19	153	Association of acute cor pulmonale (HR 4.05), RV dilatation (HR 3.33), and LVEF (HR 0.94) with mortality
Bursi et al. [55]	2022	Hospitalized patients with COVID-19	133	Lower LVEF, higher PASP, decreased TAPSE, lower TAPSE/PASP ratio in non-survivors vs. survivors, lower risk of death for every 1 mm/mmHg increase in TAPSE/PASP, TAPSE/PASP ratio cutoff for predicting mortality
Holmqvist et al. [56]	2022	ICU patients with COVID-19	132	RV dysfunction and elevated PASP were associated with a higher risk of death at 30 days
Dadon et al. [57]	2022	Hospitalized patients with COVID-19	102	An abnormal echocardiogram was associated with advanced ventilatory support, acute decompensated heart failure, myocardial injury, acute kidney injury, death, and composite endpoint myocardial injury, acute kidney injury, death, and a composite endpoint
Salem et al. [58]	2022	Hospitalized patients with COVID-19	127	Echocardiography findings were not associated with mortality or intensity of O_2_ requirement
Vieira et al. [59]	2021	Hospitalized patients with COVID-19	111	Tricuspid regurgitation velocity was related to the endpoint of renal failure, pulmonary thromboembolism, and mortality

ARDS—acute respiratory distress syndrome; CAP—community acquired pneumonia; CTPA—computed tomography pulmonary angiogram; EF—ejection fraction; HR—hazard ratio; ICU—intensive care unit; IMV—invasive mechanical ventilation; IVC—inferior vena cava; LV—left ventricle; LVDD—left ventricle diastolic diameter; LVEF—left ventricle ejection fraction; LVIDd—left ventricular internal dimension-diastole; LVSWI—left ventricle stroke work index; MEWS—modified early warning scale; OR—odds ratio; PASP—pulmonary artery systolic pressure; PE—pulmonary embolism; PH—pulmonary hypertension; PVAT—pulmonary valve acceleration time; RAID—right atrium internal dimension; RRT—renal replacement therapy; RV—right ventricle; RVIDd—right ventricle internal dimension-diastole; RVSP—right ventricular systolic pressure; SVR—systemic vascular resistance; TAPSE—tricuspid annular plane systolic excursion; Vmax—maximum velocity.

**Table 2:** Summary of the studies on the value of computed tomography angiography (CTA) in COVID-19.

Author	Year	Population	Number of Patients	Key Observations
Fauvel et al. [62]	2020	Hospitalized patients with COVID-19	1240	8.3% of patients were diagnosed with PE; 77.7% of PEs were diagnosed within the first 48 h after admission
Alaithan et al. [63]	2021	Patients who underwent CTPA to rule out PE	316, including 158 COVID-19 patients	Overutilization of CTPA in COVID-19 patients
Riyahi [64]	2021	Hospitalized patients with COVID-19	413	The difference in mortality between patients with and without PE was not significant
Hernandez et al. [65]	2020	Hospitalized patients with COVID-19	399	No significant differences (clinical, laboratory, or radiologic) between patients with and without COVID-19 were found
Loffi et al. [66]	2021	Hospitalized patients with COVID-19	333	No significant difference in mortality in patients with vs. without PE
Silva et al. [67]	2021	COVID-19 patients admitted to ED	300	Age, D-dimers, and cTnT-hs) identified as PE predictors
Gil Mosquera et al. [68]	2022	ED patients with COVID-19	274	PE was confirmed in 25.54% of patients; D-dimer > 3000 ng/mL and tachypnoea were predictive factors of PE
Planquette et al. [69]	2021	Hospitalized patients with COVID-19	269	Increased risk of PE in patients treated by IMV
Filippi et al. [70]	2021	Hospitalized patients with COVID-19	267	Non-Caucasian race and previous VTE as independent PE risk factors
Scudiero et al. [71]	2021	Hospitalized patients with COVID-19	224	Association between presence of PE and time between symptom onset and hospitalization; acute cardiac injury, D-dimer TAPSE, and PASP
Tsakok et al. [72]	2022	Hospitalized patients with COVID-19	137	Association of CTSS and ICU admission and death
Korevaar et al. [73]	2021	ED patients with COVID-19	169	Median D-dimer was significantly higher in patients with PE vs. without PE
Ippolito et al. [74]	2021	Hospitalized patients with COVID-19	170	Significant correlation between the PAO index and D-dimer level, and no significant differences between CT of laboratory findings and patients’ death
Grillet et al. [75]	2020	Hospitalized patients with COVID-19	100	Patients with PE compared with patients without PE were more frequently hospitalized in ICU and required mechanical ventilation

CAP—community acquired pneumonia; cTnT-hs—highly sensitive cardiac troponin T; CTPA—computed tomography pulmonary angiogram; CTSS—computed tomography severity score; ED—emergency department; HR—hazard ratio; ICU—intensive care unit; IMV—invasive mechanical ventilation; OR—odds ratio; PAO—pulmonary artery obstruction; PASP—pulmonary artery systolic pressure; PE—pulmonary embolism; TAPSE—tricuspid annular plane systolic excursion; VTE—venous thromboembolism.

## Data Availability

No new data were created or analyzed in this study. Data sharing is not applicable to this article.
